# Therapeutic alternatives in the management of osteoradionecrosis of the jaws. Systematic review

**DOI:** 10.4317/medoral.24132

**Published:** 2020-10-09

**Authors:** Gisela CV Camolesi, Karem L. Ortega, Janaina Braga Medina, Luana Campos, Alejandro I Lorenzo Pouso, Pilar Gándara Vila, Mario Pérez Sayáns

**Affiliations:** 1 DDS. Assistant Professor of Specialization in Oral Maxillofacial Surgery at Foundation for Scientific and Technological Development of Dentistry, University of São Paulo, Brazil; 2 PhD, DDS. Department of Stomatology, School of Dentistry, University of São Paulo, Brazil; 3 DDS. Department of Stomatology, School of Dentistry, University of São Paulo, Brazil; 4 Division of Dentistry, Mario Covas State Hospital of Santo André, São Paulo, Brazil; 5 PhD, DDS. Department of Post-graduation in Implantology, University of Santo Amaro, School of Dentistry. SaÞo Paulo, Brazil; 6 Oral medicine, Brazilian Cancer Control Institute. São Paulo, Brazil; 7 DDS. Oral Medicine, Oral Surgery and Implantology Unit (MedOralRes). Faculty of Medicine and Dentistry Universidade de Santiago de Compostela, Spain; 8 PhD, DDS. Oral Medicine, Oral Surgery and Implantology Unit (MedOralRes). Faculty of Medicine and Dentistry Universidade de Santiago de Compostela, Spain

## Abstract

**Background:**

to systematically review the literature, comparing the healing of osteoradionecrosis (ORN) among the therapeutic alternatives: surgical, pharmacological and combined.

**Material and Methods:**

The review was organized according to the PRISMA protocol with regards to the following PICO question: patients with ORN of the jaws (P=Patient); all interventions reported (I = intervention); between all therapies (C=Comparison); healing of lesions (O=outcome).

**Results:**

Surgical treatment was the most common choice (46.3%) followed by pharmacological treatment, exclusively (25.9%) or combined (26.9%). Treatment exclusively by surgical intervention seems to be most effective option, with 51.2% of the lesions healed, OR for healing of 5.7 (CI95% 1.9-16.9, *p*=0.002). Only 1 case (0.9%) corresponded to low level laser therapy.

**Conclusions:**

It seems clear that early intervention with conservative surgical combined with pharmacological methods improves the prognosis of ORN.

** Key words:**Osteoradionecrosis, radiotherapy bone necrosis, hyperbaric oxygen, pentoxifylline, teriparatide, low level laser therapy.

## Introduction

Radiotherapy (RT) alone or in combination with chemotherapy or surgery is an established form of therapy for the treatment of head and neck cancer (1). Nonetheless, it has significant limitations due to its short-term (such as mucositis, dry mouth and loss of taste) and long-term (subcutaneous soft-tissue fibrosis, neck muscle atrophy, swallowing abnormality, carotid damage, trismus, radiation caries, and osteoradionecrosis (ORN) side effects (2,3). Despite the use of 3D conformal RT (3D-CRT) and Intensity Modulated RT (IMRT), ORN of the jaws remains one of the most common resulting complications(4,5). The reported incidence of ORN in the population of irradiated head and neck patients is rather variable, ranging from 4.7% to 37.5% and it is considered a late event, with the vast majority of cases occurring in the first 3 years following treatment (6,7).

ORN can occur spontaneously due to genetic factors related to the TGF-β1 gene (8), or it can be the result of trauma (tooth extraction and denture-related irritations are common causes). Due to its low vascular nature and thicker cortical, mandibular ORN is more common than maxillary ORN (9-13). It is defined as irradiated and exposed bone tissue which fails to heal over a period of 3 months, without the presence of a residual or recurrent tumour (9,10,14,15). Although ORN can be observed without presenting bone exposure (16), normally clinically, it can range from a small area of intraoral bone exposure to extraoral fistulas and even pathological fractures. Pain, swelling, difficulties in mastication, paresthesia and facial deformities are possible sequelae of ORN and these have a significant impact on quality of life (7,17).

The pathogenesis of ORN remains unknown. Marx's initial proposal -the theory of hypoxia, hypovascularity and hypocellularity (3 Hs) leading to a non-healing wound- has recently been questioned, and likewise, it has not been supported by the results of several subsequent studies (9,18,19). In 2004, Delanian (20) proposed the radiation-induced fibroatrophic process (RIF) theory, which includes the formation of free radicals, endothelial dysfunction, inflammation, microvascular thrombosis, fibrosis, remodelling, and eventually bone and tissue necrosis.

The chosen treatment is based on the stage of the disease, as well as patient-related factors, however, the cure actually is not the desired outcome in the treatment of ORN, it is the abolition of symptoms and progression that is the goal. Several therapies have already been reported which have led to widespread opinions, nonetheless, there is still no universally accepted approach. More traditional or early-stage approaches include conservative treatments with oral hygiene control; hyperbaric oxygen (HBO) (prophylactically or therapeutically); the use of antibiotics over a variable period of time (although ORN is not an infectious process per se); and surgical debridement. Surgical management may be classified into minor and major procedures (21). In order to achieve satisfactory results, cases which do not respond to conservative treatment choices or those which present more advanced stages are treated with surgical resection, with or without the reconstruction of vascularised tissue(21). All of these treatments were guided mainly by Marx's theory (9). More recently and in light of the pathophysiology of the disease proposed by Delanian (20), pharmacological treatment with pentoxifylline-tocopherol with or without clodronate (PENTOCLO) (22), teriparatide (23) and low-level laser therapy (LLLT) (24) have been introduced.

Therefore, the aim of this paper is to systematically review the literature, comparing the healing of ORN with all the reported therapies: surgical, pharmacological and combined.

## Material and Methods

- Protocol and registration

The design of this study was registered in PROSPERO (Ref. 159983). This review was carried out following the PRISMA guidelines and according to the PICO method (25): patients with ORN of the jaws (*P*=Patient); all interventions related (I = intervention); between all therapies (C=Comparison); healing of lesions (O=outcome).

- Selection criteria, sources of information and search

We conducted a bibliographic search in PubMed, Web of Science, Scopus, LiLACS, OVID, EMBASE, Cochrane Library, Clinical Trials, the five WHO regional bibliographic databases (AIM, LILACS, IMEMR, IMSEAR, WPRIM), and the Conference Proceedings Citation Index in order to identify relevant studies on ORN of the jaws between the first records found in the database and November 2019.

Inclusion criteria: All of the articles on case series, case reports, cohort studies, and case and control studies with no language limitation were included.

Exclusion criteria: Articles which do not deal with RT-induced osteonecrosis; unavailable abstract; complete maxillectomy; other systematic reviews; studies that have not been conducted on humans.

Selection of studies: Two independent researchers, MPS and GCVC, analysed the abstracts of the articles obtained in the search which had met the search criteria, that is to say, texts that dealt with patients with ORN of the jaws and their management. Both of the researchers subsequently read the full article in order to determine whether or not it met the inclusion criteria. A third researcher, LC, acted as a mediator in the case of any disputes.

Data collection process: Data from all the articles was collected by both researchers independently (in duplicate) and this data was corroborated by the third party who acted as a mediator in case of discrepancy or lack of agreement.

- Study variables

The following information was extracted from each study: First author, year of publication, type of study, location of cancerous lesion, dose used in RT, management of the lesion (surgical, pharmacological or combined), location (maxilla, jaw), region (anterior, posterior), quantity (single, multiple), and also the time from the end of RT to the diagnosis of ORN, time until healing, maximum follow-up time, and finally whether or not there were any recurrences.

- Risk of bias

The methodological quality and the risk of bias of the included studies were assessed using the Newcastle-Ottawa scale (NOS)(26). For studies, cohorts and cases and controls, which amounted to 4.6% of the included studies, the original NOS scale was used, and for the remaining 95.4%, that is to say, the case series and case report studies, Pierson and Bradford Hills’ modified NOS scale (27) was used. This analysis was carried out independently by each of the two researchers and in the case of any disagreements the third researcher acted as a mediator.

- Statistical analysis

All of the variables were collected in a database and were analysed with SPSS v. 24.0 (IBM Inc., Madrid, Spain). Basic descriptive statistics were used for the univariate description, these included the mean, standard deviation, frequency and percentage. The relationship between the different categorical variables and healing was evaluated using Pearson's Chi-square. The relationship between the healing and the type of treatment and the quantitative variables was studied by using the ANOVA test to compare the means. The influence of the treatment type on the progression of ORN was assessed by using a univariate logistic regression analysis. The significance level was established at *p* ≤0.05.

## Results

**Figure 1:**
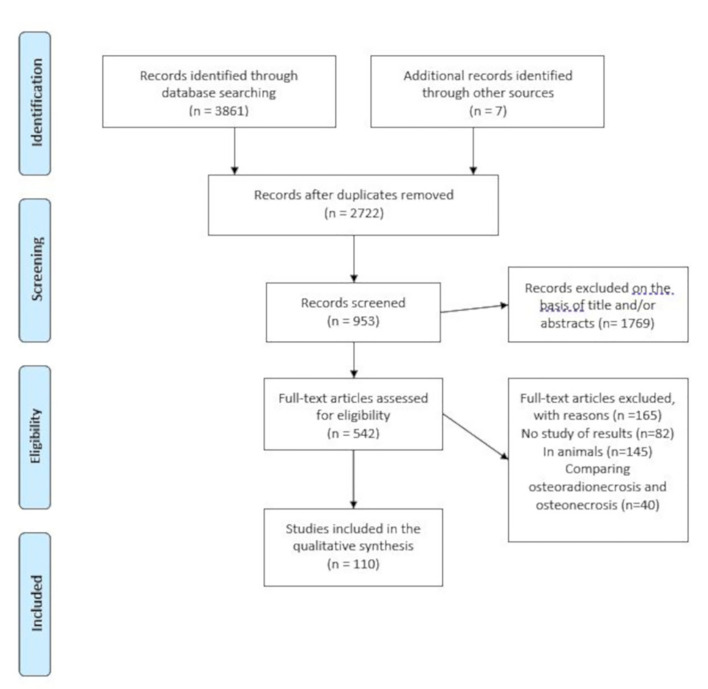
PRISMA flow diagram.

The search process involved a total of 3,861 articles. After removing duplicates, 2,722 articles remained; of these 1,769 were subsequently excluded because they did not meet the inclusion criteria (Fig. 1). After fully reading the 542 articles, it was determined that 110 studies m*et al*l of the inclusion criteria and these were included.

Four of the articles were rated as high quality (3.6%), 103 as medium quality (93.6%), and 3 as low quality (2.7%) (Table 1). The summary of the data of all of the patients that was extracted from the studies is depicted in Table 2, and the full descriptive results can be found in Table 3.

**Table 1:**
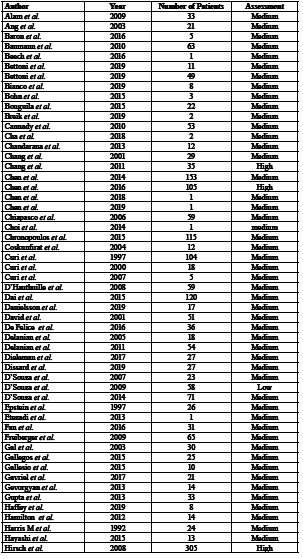
Classification of the studies in terms of risk of bias according to the NOS scale.

**Table 1 cont.**
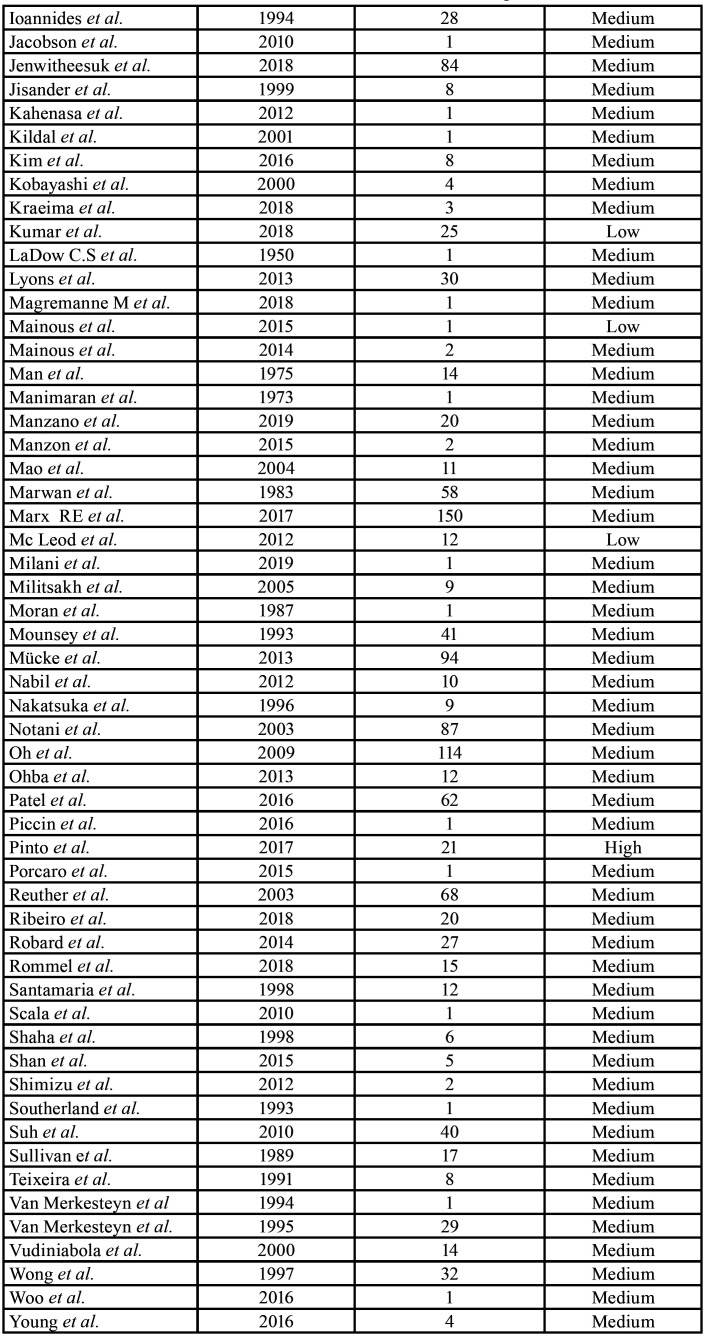
Classification of the studies in terms of risk of bias according to the NOS scale.

**Table 2:**
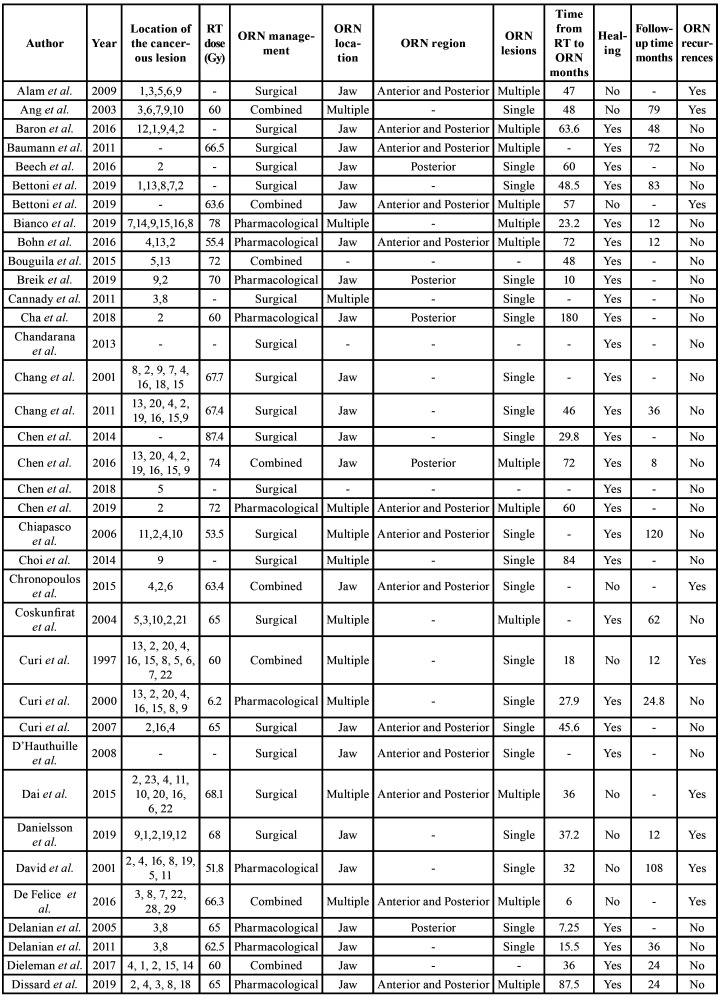
Descriptive summary of all of the articles.

**Table 2 cont.**
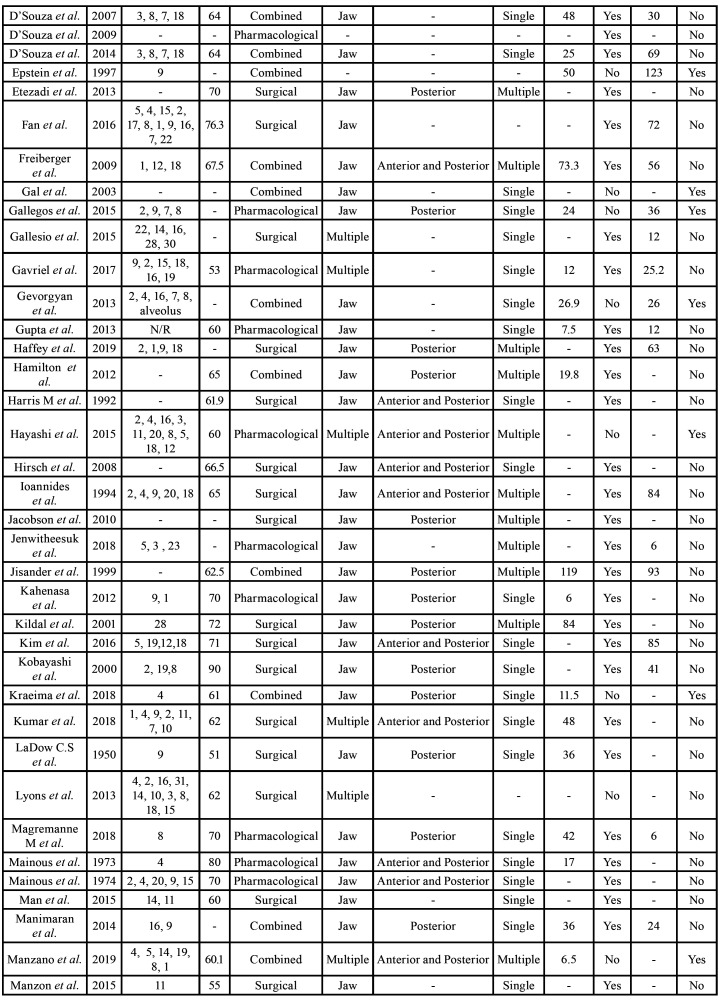
Descriptive summary of all of the articles.

**Table 2 cont.**
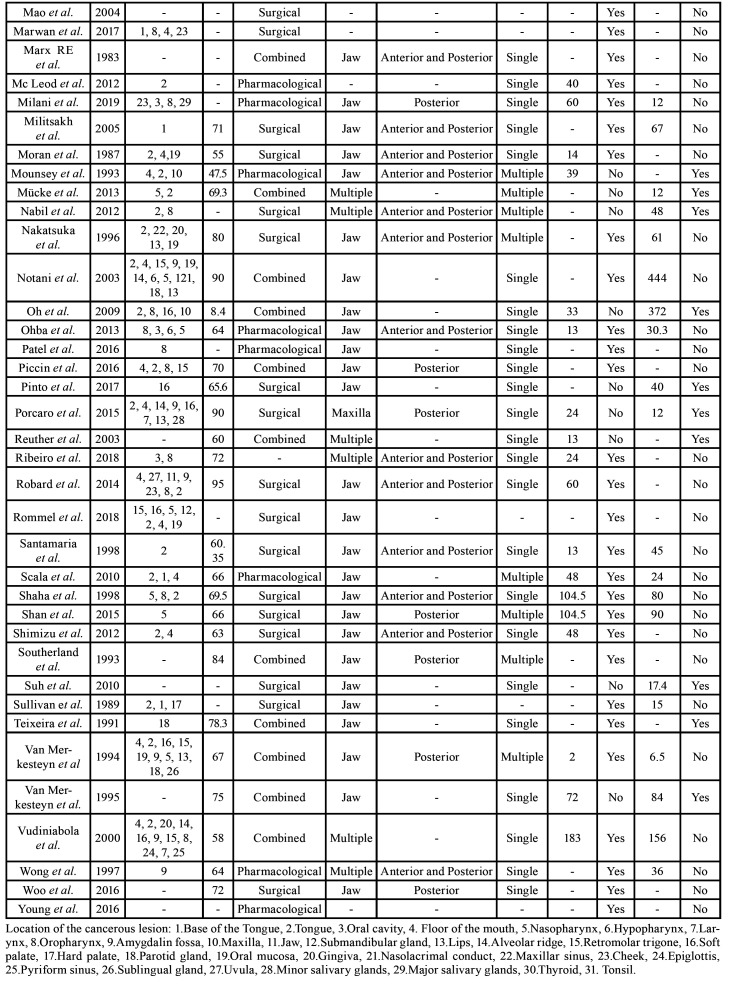
Descriptive summary of all of the articles.

**Table 3:**
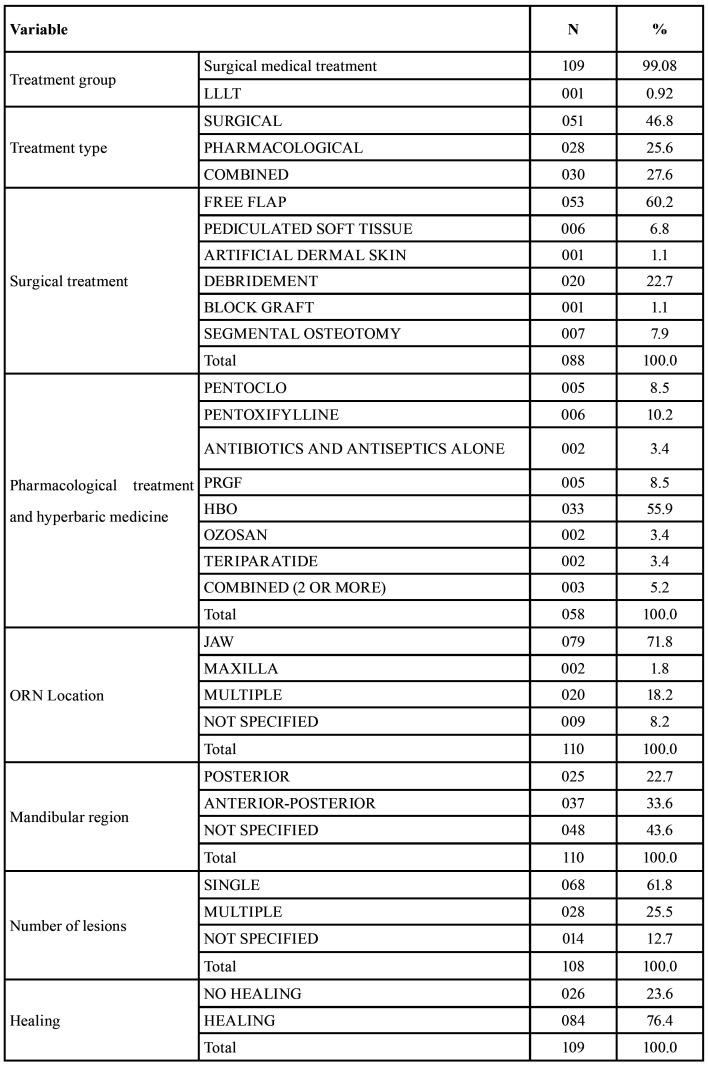
Descriptive summary of extracted categorical variables.

With regards to the characteristics of the ORN found, 9 (8.2%) of the articles did not specify the location of the lesion. Out of the 81 articles with a single lesion, 2 of them presented in the maxilla (1.8%), 79 in the jaw (71.8%), and 20 (18.2%) presented in both jaws. With regards to the number of lesions, 61.8% of the articles described single lesions, 25.5% described multiple simultaneous lesions, and in 12.7% of the articles this was not specified. In 22.7% of the cases, the lesions appeared solely in the posterior region, however in 33.6% of the cases these appeared both in the anterior and posterior sectors.

As far as the mean onset time, there was a significant variability in with a range from 2 months to 183 months, however the mean was 45.7 months (SD=36.2), that is to say 3.8 years. Evidently the appearance of the lesions depends on the maximum follow-up time, which, in this systematic review was broad and variable, ranging from 6 to 444 months, with a mean of 58.9 months (SD=76.5).

In terms of the therapeutic alternatives used, the surgical treatment was the most common choice representing 45.5% of cases, and pharmacological treatment, exclusively or combined, was the least common, with 26.1% and 28.4% of cases respectively. Only one study, that is to say 0.90% corresponded to the treatment of ORN by LLLT. Radical surgical treatment with free flap was the most used surgical alternative in 60.2% of the cases, followed by debridement (curettage and/or sequestrectomy or marginal resection) in 22.7%. In terms of exclusively pharmacological treatments, HBO accounted for 58.3%, followed by the use of pentoxifylline, with or without clodronate in 21.6% of the cases. The systematic review shows an overall healing of 77.2% of the lesions.

The healing of the ORN lesions is understood as the absence of relapse during the follow-up period, which as shown before, is very variable. This healing appears to vary depending on the type of treatment performed. Out of 88 cases which were treated by surgical intervention, only 73.7 % of the cases were cured, and likewise, 70.0% of the 60 cases, which were treated by pharmacological means were cured. Broadly speaking, treatment exclusively by surgical intervention seems to be effective option, with 51.2% of the lesions healed, whereas only 28.6% of the lesions of patients who were treated exclusively by pharmacological means, and 17.9% of the lesions in patients who underwent combined medical-surgical treatment (*p*=0.002) were healed. In the study conducted with LLLT therapy combined with antimicrobial photodynamic therapy (aPDT), 20 patients were treated and 100% of the patients were cured.

Statistically significant differences between healing and the type of surgical treatment were not observed, however, as we can see in Table 4, statistically significant differences were observed when using pharmacological treatment, Table 4. Pentoxifylline with/without clodronate made a major and significant contribution to the healing in 84,6 % of the cases where was used, with HBO healed in 62.8 % of the cases, whereas other alternatives, such as the exclusive use of antibiotics/anti-inflammatories/antiseptics failed in 100 % of the patients (*p*=0.043). By performing a binomial logistic regression analysis, we verified that the type of treatment is the only statistically significant factor related to healing. Therefore, taking the combined medical-surgical treatment as a reference, exclusive surgical treatment shows an OR for healing of 5.7 (CI95% 1.9-16.9, *p*=0.002) and 5.7 for pharmacological treatment (CI95% 1.5-20.2, *p*=0.009). Given that only one study was treated with LLLT, this has been excluded from the equation.

**Table 4:**
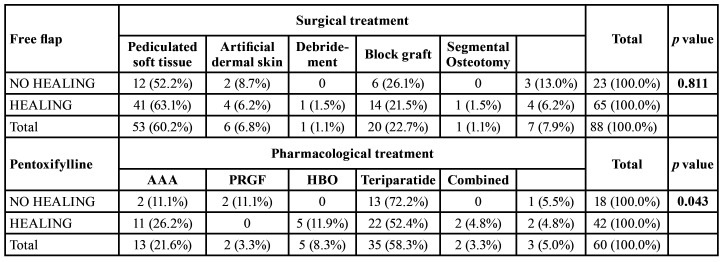
Comparison of the healing process according to the different types of treatment gathered for ORN. AAA (Antibiotics, anti-inflammatories, antiseptics); PRGF (platelet rich growth factor).

## Discussion

ORN is a serious complication which is difficult and expensive to treat (21). In order to manage the disease in its early stages, the treatment must be conservative. The authors recommend oral hygiene, optimisation of the nutritional condition and a multidisciplinary management, which includes minor surgery, how the dental extraction, or debridement of the necrotic tissue and antibiotics (28).

In the 1960s, after its implementation by Marx (9), HBO began to be used as an additional treatment for ORN, as a complement for soft tissue flaps and in the management of radiated tissues. Although HBO initially showed promising results in the treatment of ORN (11), today’s literature shows very disparate results in the use of this technique (29).

In the advanced stages or recurrences of the disease, a surgical reconstruction of the jaw is performed by means of the surgical resection and immediate transfer of the tissue to the disease, especially in stage III (30). The reconstruction of a free flap in the radiated jaw is difficult. The identification and dissection of the receiving vessels can be arduous and it requires for vessels to be selected from outside of the radiated field, generally from the contralateral neck (13).

Furthermore, it is an expensive procedure, due to hospital stay (21). Recently factors like appearance, swallowing, and chewing that interfere with the quality of life were analyzed and showed that the approach with adequate debridement, resection, and reconstruction may greatly improve QOL (31). The surgical treatments identified in the studies include sequestrectomy and debridement (32), free flap (33), pediculate soft tissue (34) and block grafting (35).

In this review, 21.6% of the studies presented ORN cases, which were treated with pentoxifylline and PENTOCLO. This management is used in both the early and advanced stages of the disease. The combined medical therapy showed a recovery rate of 88.9 % in the 13 presented studies, and in just 11.1 % of them (2 studies), the disease progressed and subsequent surgery was necessary for healing. Some of these studies presented patients whose recovery had already failed with other conservative therapies, such as the study conducted by Delanian (22), in which 16 out of 18 patients completely recovered and, out of these, 14 were fully recovered within 7 months. In 2011 (36), a subsequent study conducted by the same researchers on refractory ORN of the jaw treated by means of HBO and surgical intervention, studied the combination of pentoxifylline and vitamin E, together with clodronate, antibiotics and steroids as treatment. All of the patients (100 %) presented with a complete regression of the exposed bone and were fully recovered within 2 years after treatment, with 50 % of the patients recovering in just 6 months.

In research performed by D'Souza (7), the results of ORN patients who had received medical treatment with pentoxifylline, tocopherol and doxycycline were compared with those of patients who had been treated with HBO. 25% and 51% of the patients respectively showed a progression of the disease and required free flap reconstruction. Furthermore, in the group of patients that received medical treatment there were no recurrences of ORN following the resection and the free flap reconstruction, in comparison with a 20% recurrence in the group treated with HBO. This confirms the current understanding of the pathophysiology of ORN based on the fibrosis induced by radiation.

Recently, other alternatives for the management of ORN have been discussed in the literature, and these include plasmatic factors modified in all their versions (PRGF, PLT-gel, L-PRF), Teriparatide and LLLT. With regards to plasma rich in growth factors (PRGF), its use was suggested following reports in which it was demonstrated that its application as filling material in surgeries and pre-prosthetic implants presented excellent adjuvant and regenerative proprieties (37). The RIF process reduces the level of expression of the transforming growth factor beta (TGF-β). The use of PRGF formulations is based on the premise that the growth factors contained in platelet granules, which are released after activation are beneficial to improving the tissue regeneration (37). In a study, which was performed by Gallesio on 10 patients (38), on day 14 after surgery, the treated area presented complete wound closure.

Cha (23), presented a study in which Teriparatide -a recombinant human parathyroid hormone- was used, demonstrating its beneficial effects on bone regeneration of ORN of the jaw in advanced stages. However, the studies performed on rat models have shown a theoretical risk of osteosarcoma, therefore confirming the need for further studies (39).

The only LLLT report found in our review dated back to 2018 (24). The effectiveness of LLLT is supported by studies in which its effects on the healing process of the oral mucosa are highlighted. These studies have also demonstrated that it minimises the exudative phase, boosts healing and leads to the proliferation and transformation of fibroblasts and myofibroblasts that help in tissue repair, due to the release of growth factors (40). Ribeiro (24) presented a protocol for management with LLLT, in which the 20 treated patients presented with the pathology in early to advanced stages. 100 % of the reported cases were healed with no recurrence during the two follow-up years. This therapy is also non-invasive, atraumatic and no significant associated adverse effects have been reported in the literature.

Among the limitations to this systematic review, it is important to mention that it mostly consists of a retrospective group of cases and case reports, therefore meaning that their heterogeneous nature and the absence of randomised trials is a limiting factor. As a consequence of these disadvantages, the possibility of carrying out a more objective analysis in which more powerful conclusions are drawn would prove challenging.

The results obtained out of all of the different treatments proposed for ORN, seem to indicate that the combined surgical and / or pharmacological treatment (PENTOCLO), is the treatment of choice and offers better healing rates. In case of recurrence, there is some evidence that resection surgery and reconstruction may also be considered, respecting the particular circumstances in which each should be used. What seems clear is that early intervention with conservative surgical and pharmacological methods improves the prognosis of ORN. In an attempt to expand less invasive treatment methods, we suggest more studies for conservative surgical management of hard tissue associated with LLLT therapy, based on controlled clinical studies, with well-distinguished control groups are necessary in order to establish a more efficient therapeutic pattern.
